# Preparation of a Novel Resin Based Covalent Framework Material and Its Application in the Determination of Phenolic Endocrine Disruptors in Beverages by SPE-HPLC

**DOI:** 10.3390/polym13172935

**Published:** 2021-08-31

**Authors:** Yunjie Ma, Yang Ruan, Xin Gao, Hang Cui, Wei Zhang, Shaoyan Wang

**Affiliations:** School of Chemical Engineering, Liaoning Provincial Key Laboratory of Fine Separation Technique, University of Science and Technology, Liaoning, Anshan 114051, China; cda0429@163.com (Y.M.); realruanyang@163.com (Y.R.); askdgx@163.com (X.G.); cuihang703@163.com (H.C.)

**Keywords:** resin based covalent organic framework materials, phenolic endocrine disruptors, solid phase extraction, trace analysis

## Abstract

A new type of economical covalent organic framework material(COF), namely resin based covalent organic framework material, was prepared by combining resin and covalent organic framework material by hydrothermal synthesis, which was based on the preparation of traditional COF material(TpBD COF). The properties of the material and covalent organic framework material were compared in the way of characterization, and the possible reaction mechanism was analyzed. The solid phase extraction separation (SPE) ability of this material for four kinds of phenolic endocrine disrupting compounds (bisphenol F, bisphenol A, octylphenol and nonylphenol) in beverage samples was investigated. The results showed that the prepared COF materials had abundant internal channels, ordered structure, large specific surface area (TpBD COF: 814.6 m^2^/g and resin based COF: 623.9 m^2^/g) and good thermal stability (pyrolysis temperature was 443 °C and 437 °C, respectively). Solid phase extraction experiments demonstrated that the two COF materials as adsorbent of solid phase extraction column had ideal adsorption separation effect and good anti-interference ability, and had strong anti-interference ability. The SPE effect was superior to the traditional solid phase extraction column. The precision RSD of this method was less than 3%. This SPE method had high recovery and could be reused (carbonated beverage: 98.18–102.18% and beverage: 98.52–101.79%), In addition, the recovery of the material did not change significantly in the 50 cycles of solid phase extraction, indicating that the material had good stability and could be reused, which could meet the requirements for the detection and analysis of trace pollutants in environmental samples. The resin based COF material prepared in this study could reduce the cost of monomer uses and provide a possibility for its industrial production. At the same time, as an efficient SPE adsorbent, it also provided a new research scheme for the enrichment of trace phenolic endocrine disruptors in beverage samples.

## 1. Introduction

Phenolic endocrine disruptors (EDCs) have attracted widespread attention because they mimic the effects of endogenous hormones and interfere with the function of endocrine organs, thus causing serious threats to human health (including reproductive dysfunction, birth defects, metabolic disorders and some malignant tumors [1,2]). Among them, bisphenol A (BPA) and bisphenol F (BPF), as important intermediates, are mostly used in the production of polycarbonate plastics and epoxy resins, ref. [3,4,5,6] while nonylphenol (NP) and octyl phenol (OP) are widely used in the production of plastic bags, plasticizers, detergents, pesticides, etc [7,8,9,10]. These EDCs widely exist in the environment, causing potential harm to human health [11,12], and the amount of these disruptors is very small in environmental samples. Solid phase extraction (SPE) enrichment technology has become a powerful tool for separating and concentrating to trace analytical substances [13] by virtue of its characteristics of less solvent consumption, simple operation, high selectivity, good reproducibility and strong anti-interference ability, and has been widely used in drug analysis [14], toxicant analysis [15], environmental detection [16] and other aspects. The key of solid phase extraction is the selection of adsorbents. Generally, porous materials with large specific surface area and stable chemical properties are selected. Covalent organic frameworks (COFs), as a new type of porous material, have a larger specific surface area and porous structure, and good thermal stability. It has great potential as an ideal adsorbent for solid phase extraction pretreatment and enrichment of organic compounds [17,18,19].

In this paper, the traditional adsorption resin and covalent organic framework materials were combined by hydrothermal method. A new type of economical resin based covalent organic framework material was prepared, and four EDCs were enriched by solid phase extraction (SPE) to study the contents of endocrine disruptors in beverage samples. Scanning electron microscopy (SEM), surface area measurement, thermogravimetric analysis (TGA), infrared spectroscopy (IR) and X-ray diffraction (XRD) were used to analyze the structure and basic physical properties of the two COF prepared. Meanwhile, the SPE effect was compared with the COF-filled SPE column and commercial SPE column. The results indicated that this novel COF material could be used as a reference for the enrichment and detection of EDCs in environmental samples.

## 2. Materials and Methods

### 2.1. Reagents and Instruments

1,3,5–Triformylphloroglucinol (Tp), benzidine (BD), diethylbenzene and sodium hydroxide, Alpha Chemical (Zhengzhou, China).; tritoluene, octylphenol (OP) and nonylphenol (NP), Aladdin (Shanghai, China); bisphenol A (BPA), Shanghai Xushuo Biotechnology Co., Ltd. (Shanghai, China); bisphenol F (BPF), Bide Pharmatech Ltd. (Guizhou, China); styrene, benzoyl peroxide (BPO), toluene, chloroform, liquid paraffin, gelatin, methanol, acetic acid and ethanol, Sinopharm Chemical Reagent Co., Ltd. (Shanghai, China); all the above reagents were analytically pure. Strata-X, Strata-X-CW, Strata-X-C and Strata-X-A, Phenomenex (Tianjin, China). 

THZ-82A Constant Temperature Oscillator, Jintan Chengdong Xinrui Instrument Factory (Jiangsu, China); DHG type vacuum drying oven, Shangyu Huyue Equipment Factory (Zhejiang, China); FTIR-1500 Fourier transform infrared spectrometer (FTIR), Mettler Toledo Instruments Co., Ltd (Shanghai, China); Evo18 field emission high resolution scanning electron microscope (SEM), Zeiss AG, (Shanghai, China); NOVA 3200E specific surface area analyzer, Kantakmer Instrument Trading Co., Ltd. (Shanghai, China); LC-10AT high performance liquid chromatograph, Shimadzu Co., Ltd (Shanghai, China); B. V.Epyrean X-ray powder diffractometer (XRD), Panaco, Netherlands.

### 2.2. Experimental Methods

#### 2.2.1. Preparation of TpBD COF Material

First, 15 mL ethanol solution containing 0.3 mmol Tp and 5 mL BD solution containing 0.45 mmol Tp were mixed in a 100 mL flask and stirred continuously for 15 min at room temperature to obtain a uniform yellow solution. The mixture was refluxed at 80 °C and protected by nitrogen, and the mixed solution changed from yellow to light brown. Then the mixed solution was strongly stirred for 3 h under refluxing, and the obtained solution was centrifuged (10000 r/min, 5 min), After centrifugation, the product was dried in a vacuum drying oven at 30 °C for 24 h to obtain the dried TpBD COF material [20]. 

#### 2.2.2. Synthesis Mechanism of TpBD COF Material

**Figure 1:**
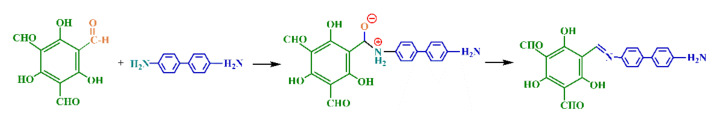
Mechanism diagram of TpBD COF material synthesis.

As shown in Figure 1, the synthesis mechanism diagram of TpBD COF material. The COF material prepared in this experiment was prepared for Tp and BD as raw materials and hydrothermal synthesis of aldehydes and amines. –CHO in Tp and NH_2_ in BD were condensed to prepare and form intermediate products.The resulting hexagonal ring structure was showed in the figure above, and the size of the structure was directly related to the amount of raw materials added.

#### 2.2.3. Preparation of Resin Based COF Material

Weighed 0.219 g of resin powder [21], took 0.1262 g of Tp into 30 mL of anhydrous ethanol and 0.1658 g of BD into 10mL of anhydrous ethanol, stirred them, respectively, until dissolved, and stirring continued for 15 min. The mixture was poured into a flask in a water bath at 80 °C for heating, reflux condensation and nitrogen was passed through for protection. After heating and reaction for about 24 h, the color of the mixture solution changed from yellow to light brown. After stirring for 3 h, the mixture was centrifuged at 10,000 r/min for 5 min, and the separated solid substances were extracted by DMF and anhydrous ethanol for 50 min, respectively. Finally, the obtained solids were put into a vacuum drying oven at 80 °C for drying.

#### 2.2.4. Synthesis Mechanism of Resin Based COF Material

**Figure 2:**
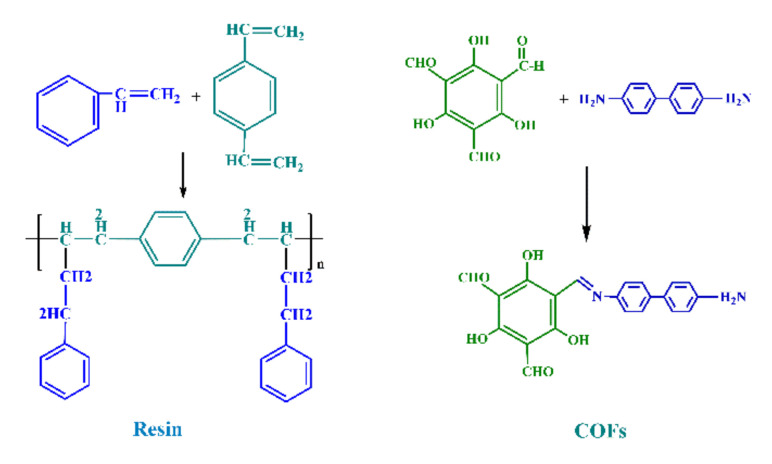
Reaction process of two materials.

**Figure 3:**
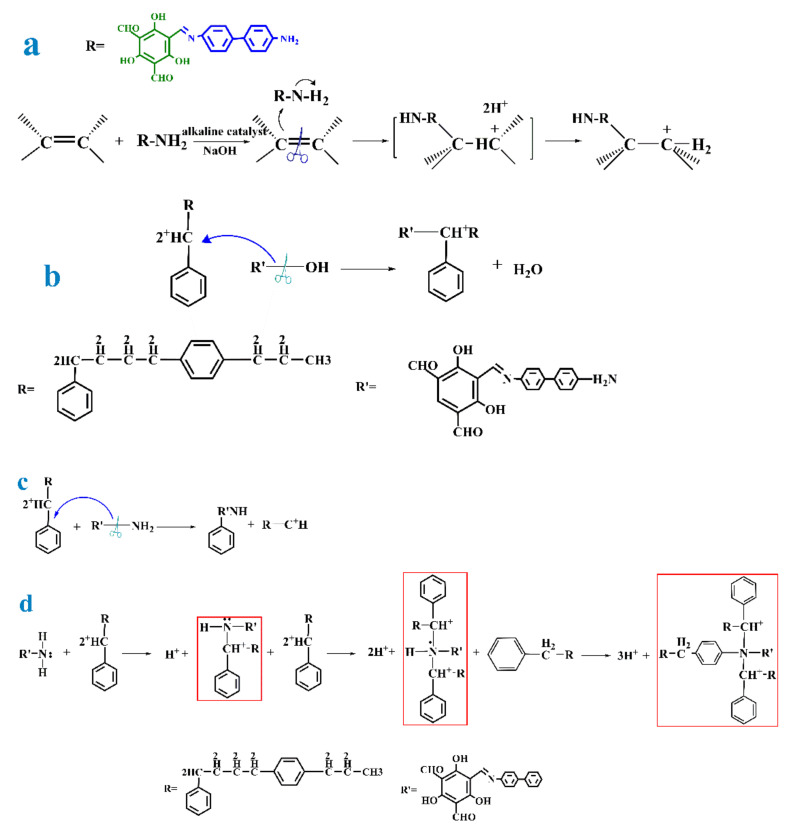
(**a**) When the resin material has free C double bond, C=C double bond and TpBD COF reaction process. (**b**) The reaction process in which the –CH_2_– in the resin material is dehydrated with the –OH in the COF material. (**c**) Reaction process of –NH_2_ fracture in COF with –CH_2_– in resin. (**d**) Alkylation between –NH_2_ and –CH_2_– in the resin and the introduction of carbonaceous substances on *N* atoms.

As we can see from Figure 2 and Figure 3, the reaction processes and reaction mechanisms of the resin and COF are shown, respectively. The synthesis reactions of the two materials were polymerization reaction and aldehyde amine condensation reaction, respectively. Figure 3a shows the reaction process of C=C double bond and TpBD COF when the resin material had a free C double bond. The reaction was an addition reaction of C=C double bond and –NH_2_, and the addition reaction was one of the typical reactions to increase the chain length of reactants. The polymer length was also related to the content of C=C double bond and -NH_2_. Figure 3b–d shows the possible reaction mechanisms of –CH_2_– in the resin material with –OH and –NH_2_ in the COF material when the C=C double bond did not exist and the monomers of the two materials were all reacted. Figure 3b shows that -CH_2_- in the resin material dehydrates with -OH in the COF material, which made the two materials combine. The length of the final product was related to the content of –CH_2_– and –OH. The reaction process in Figure 3c is that –NH_2_ in COF breaks and reacts with –CH_2_- in resin to form the final product through the continuous addition of reaction monomer. Figure 3d shows the alkylation reaction between –NH_2_ and –CH_2_– in the resin and the introduction of carbon containing substances on the *N* atom. It could be seen that the molecular structure of the product prepared by this synthesis mechanism was larger than that of the product synthesized by other synthesis mechanisms, but the dosage of the resin also increased significantly. The length of products in Figure 3c,d were related to the content of –CH_2_– and –NH_2_. The above analysis was the possible reaction mechanism in the preparation process of resin based COF. Combined with the specific preparation process used in this experiment, it could be concluded that the synthesis mechanism in Figure 3d was the most likely, and the cost of preparation process and material usage under this mechanism was the lowest, which was more suitable for practical production application.

#### 2.2.5. Solid Phase Extraction Experiment

Environmental samples came from commercially available carbonated and vitamin beverages. Standard samples of four phenolic interferents were obtained by dissolving a certain amount of bisphenol A, bisphenol F, octyl phenol and nonylphenol in acetonitrile solution, and the analytical conditions of these compounds were as follows.

Shimadzu LC-10 was used for HPLC analysis. The detection wavelength was 228 nm. Chromatographic analysis was performed on an Agilent XDB-C18 (4.6 mm × 250 mm, 5 μm) column at 35 °C with an injection volume of 20 μL. The mobile phase consisting of solvent A (water) and solvent B (acetonitrile) was delivered at a low flow rate of 1 mL min^−1^. The program was run in a linear gradient:

0–3 min, the ratio of B in mobile phase was 60%;
3–10 min, the proportion of B in the mobile phase increased to 70%;
10–20 min, the proportion of B in the mobile phase increased to 80% and kept for 5 min;
After 25–30 min, the proportion of B in the mobile phase was reduced to 60% and kept for 30–40 min.

**Figure 4:**
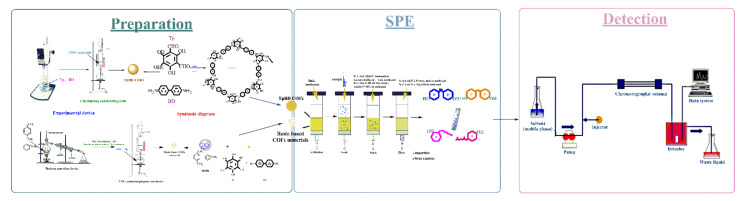
Solid phase extraction flow chart.

A certain amount of TpBD COF or resin based COF material were, respectively, filled into the empty SPE column tube which had been put into the gasket, and another spacer was lightly pressed on the material after being laid to assemble the COF-filled SPE column. Four types of SPE column (X, X–CW, X–A, X–C) were used as the control. The SPE schemes of the two self-made SPE columns and the two types of SPE columns on the market were showed in Table 1 and Figure 4.

#### 2.2.6. Characterization of Materials

The morphologies of the two COF materials were analyzed by scanning electron microscope, and the functional groups were analyzed by infrared spectroscopy (wavenumber range: 500~4000 cm^−1^). The structural characteristics of the materials were analyzed by X-ray diffraction, and the pore structure and specific surface area of the materials were obtained by specific surface area adsorption measurement method.

## 3. Results and Discussion

### 3.1. Morphology Analysis of Materials

**Figure 5:**
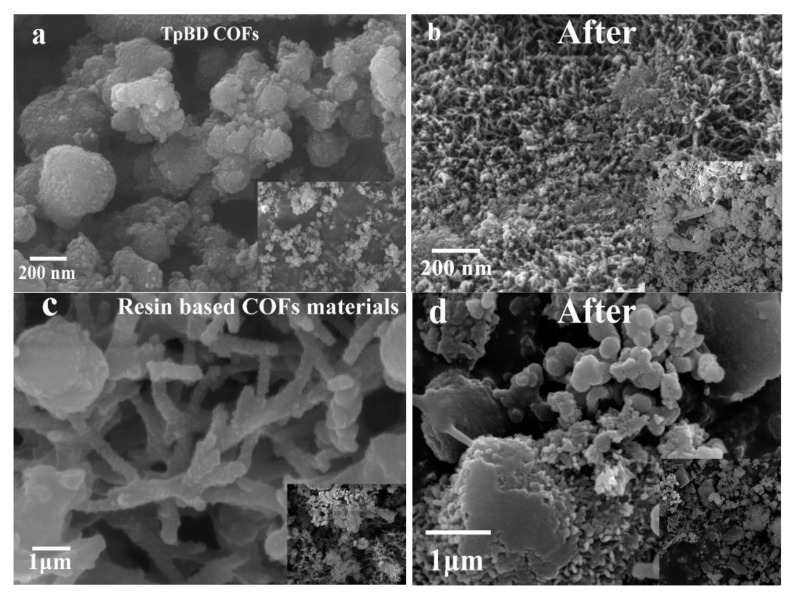
(**a**) SEM figure of TpBD COF before solid phase extraction. (**b**) SEM figure of TpBD COF after solid phase extraction. (**c**) SEM figure of resin based COF before solid phase extraction. (**d**) SEM figure of resin based COF after solid phase extraction.

Figure 5 shows the appearance and internal structure of the two materials before and after SPE, which indicated the surface of TpBD COF was irregular spherical with large pore size. The results proved that there was more reticular pores in the resin based COF, and the pore structure of the resin based COF was more abundant than that of the non-bonded COF, which indicated that the resin based COF were successfully bonded. At the same time, both materials had dense porous structure. These complex pore structures provided abundant adsorption space and large adsorption surface area for the adsorption of target substances. Both of these materials had the ability to be used as solid phase extraction enrichment fillers. In addition, the specific surface area measurement results of the two materials (Table 2) also verified the above analysis results from another point of view. In addition, it was found that the specific surface area of the new material decreased slightly after adding resin. The reason was that the added resin material had a smaller specific surface area than the COF material, so the specific surface area of the new material was reduced. After adsorbing the target material, the pore size of the two materials decreased, and residual target material was found on the surface. The results showed that the particle size of the new material was larger after adding resin. It was found that the final product quantity of resin based COF was four times that of TpBD COF. Therefore, this method not only had a breakthrough in the particle size of raw materials, but also greatly reduced the preparation cost, which was more suitable for industrial production.

### 3.2. Thermogravimetric Analysis of COF Materials

**Figure 6:**
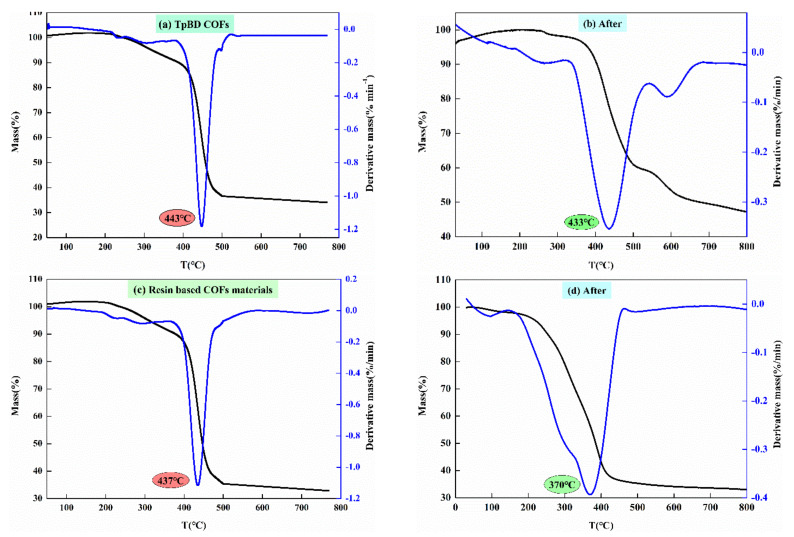
(**a**) Thermogravimetric curve of TpBD COF before solid phase extraction. (**b**) Thermogravimetric curve of TpBD COF after solid phase extraction. (**c**) Thermogravimetric curve of resin based COF before solid phase extraction. (**d**) Thermogravimetric curve of resin based COF after solid phase extraction.

The thermogravimetric curves (Figure 6) of the two kinds of COF show that the mass of COF decreased slightly at the initial stage of temperature increased, which was the result of weight fluctuation caused by incomplete removal of deep bound water in the materials; as the temperature increases, the two kinds of COF began to decompose. Taking TpBD COF material as an example, with the increase of temperature, the mass of TpBD COF material gradually decreased, and the peak values appeared at 443 °C, indicating that the COF were completely decomposed at 443 °C. The reason was that with the increase of temperature, the van der Waals force between the layers in the material structure was destroyed, the hydrogen bond between the hydroxyl group and aldehyde group in Tp structure were broken, the amino group in BD structure was pyrolyzed and the carbon chain structure was pyrolyzed continuously at 443 °C. The decomposition temperatures of TpBD COF material and resin based COF were 443 °C and 437 °C, respectively. After solid phase extraction, The pyrolysis temperatures of the two materials were 433 °C and 370 °C, respectively. The thermal stability of TpBD COF material was better than that of resin COF, but both COF showed good thermal stability, especially in the room temperature range of solid phase extraction.

### 3.3. Infrared Analysis of Materials

**Figure 7:**
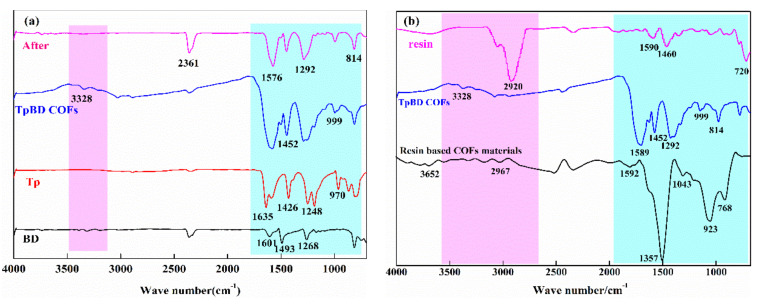
(**a**) Infrared spectra of TpBD COF. (**b**) Comparison of infrared spectra of three materials.

Figure 7 shows the infrared spectra of TpBD COF and resin based COF before and after preparation. Figure 7a shows the infrared spectrum of TpBD COF. As we can see from the figure, after the synthesis of the material, the C–H functional groups that originally existed at 814 and 999 cm^−^^1^ changed to a certain extent, which was due to the induced effect of the C–H bonded during the reaction. When the chemical bond of Tp and BD of raw materials was combined, the polarity of the bond would change, the density of electron cloud would change and the dipole would change accordingly, resulting in the change of C–H absorption peak. The absorption peak of C–N bond at ∼1452 cm^−1^ became larger after COF material was synthesized. The reason for this phenomenon was that nitrogen atom had lone pair electrons, which could conjugate with adjacent unsaturated groups and produced a mediation effect. This effect could increase the vibration wave number of the chemical bond to which it was connected, thus resulting in greater spectral absorption. The infrared spectrum was redshifted at ∼1576 cm^−1^. It was considered that the bond of the ring after the reaction had a negative effect, the larger the angle is, the less the sorbital composition of the double bond carbon outside the ring was, and the energy required for the expansion and vibration of the double bond would be reduced, so the wave number would be reduced. The infrared spectrum showed a certain degree of blue shift at the N–H functional group at ∼3328 cm^−1^. The analysis proved that during the COF synthesis, the N–H bond of BD was constantly connected, and the bond forced constant of the group becomes larger, so the stretching vibration frequency of the group increased, resulting in a certain degree of blue shift. In addition, by comparing the infrared spectra of TpBD COF prepared before and after solid phase extraction, it could be seen that the characteristic peak did not change, indicating that the COF prepared by hydrothermal synthesis was stable.

Figure 7b shows the comparison of infrared spectra of resin based COF, resin and TpBD COF. After synthesis, the peaks within 783 cm^−1^ and 923 cm^−1^ were C–H plane of the outer and inner bending vibration absorption. It was found that the absorption peaks of resin based COF were changed to a certain extent with the single resin and COF materials. The analysis proved that the C–H bond was induced during the reaction, and alkyl was the electron donor group. When the chemical bond of the raw material was combined, the polarity of the bond was transformed, the electron cloud density was changed and the dipole was also changed, which changed the C–H absorption peak; another possibility was that the bond length of the C–H bond outside the ring had a certain change due to the change of s orbital composition, and the stretching vibration wave number changed; the C–N vibration absorption peak was at ∼1043 cm^−1^. Compared with the COF material, it could be found that the absorption peak at this place was significantly strengthened. The reason for this phenomenon may be that nitrogen atoms had lone electron pairs, which could be conjugated with adjacent unsaturated groups and produced a mediation effect. This effect could increase the vibration wave number of self connected chemical bonds, resulting in larger absorption peak area. At ∼1592 cm^−1^, the skeleton vibration V_C=C_ absorption of benzene ring appeared in resin based COF. The infrared spectra of the other two materials showed that the other two materials move to a certain extent in this region. The reason might be that after the reaction, the bond angle of the ring increased, the s-orbital composition of the outer double bond carbon decreased and the energy required for the stretching vibration of the double bond reduced. The characteristic peak at ∼2967 cm^−1^ was the asymmetric stretching vibration of –C=C=C–, –C=C=O– cumulative double bond. Compared with the characteristic interval of the other two materials, it was found that the characteristic peak was unique to the new material and resin, and the absorption peak strength of the new material had a certain weakening in this interval. The analysis demonstrated that the chemical bond reacts with other chemical bonds of COF during the synthesis process, and the chemical bond broke, which was further verified by the significantly enhanced strength of the characteristic peak of C–H in-plane bending vibration absorption at ∼1357 cm^−1^. The absorption peak of resin based COF at ∼3652 cm^−1^ was N–H stretching vibration, which indicated that the characteristic peak was unique to COF and new materials, and further indicated that the bonding of resin and COF was successfully realized by this experimental method. In comparison, it was found that there was a certain degree of blue-shift in this region and the vibration frequency increased in this region. The analysis showed that when the resin based COF were synthesized, the C–H bond of Tp and the N–H bond of BD were continuously connected, and the bond force constant of the group increased, so the stretching vibration frequency of the group increased, leading to a blue shift.

Through the infrared spectrum analysis of the material, the results showed that the resin based COF material had been successfully synthesized, and the content of *N* in the resin based COF material was higher than that of TpBD COF material. According to the analysis, the SPE mechanism of the new resin based COF material was mainly due to the interaction of hydroxyl group, ester group, cyanide group and double bond.

### 3.4. X-ray Diffraction Analysis of Materials

**Figure 8:**
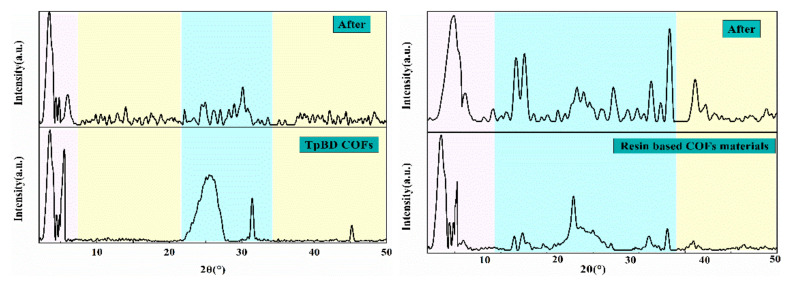
XRD patterns of the materials.

In order to further understand the crystallographic properties of the composite materials, XRD was used to test the composite materials. Figure 8 shows the XRD patterns of TpBD COF and resin based COF. The intense peaks are at ∼3.3°(2θ) for the (110) reflection and ∼6.1°(2θ) for the (200) reflection, respectively, indicating that the material has been synthesized successfully. Moreover, the intensity of diffraction peak of COF combined with resin changed to a certain extent, but the characteristic intense peak of TpBD COF appeared at the same position, indicating that COF was successfully loaded on the resin surface. During the comparison before and after solid phase extraction, it was found that although the shape of the diffraction peaks of the two materials changed, the characteristic intense peaks between 2°(2θ) and 10°(2θ) remained, demonstrating that the crystallization effect of the two materials was good. 

### 3.5. Comparison of Solid Phase Extraction Performance

#### 3.5.1. Determination of Detection Limits of Phenolic Substances

Phenolic solutions to different concentration gradients were prepared, and four phenolic solutions with different concentrations were detected and analyzed to obtain the correlation coefficient, and then the detection limit was determined by three times of the signal-to-noise ratio (SNR). The results are showed in Table 3.

**Figure 9:**
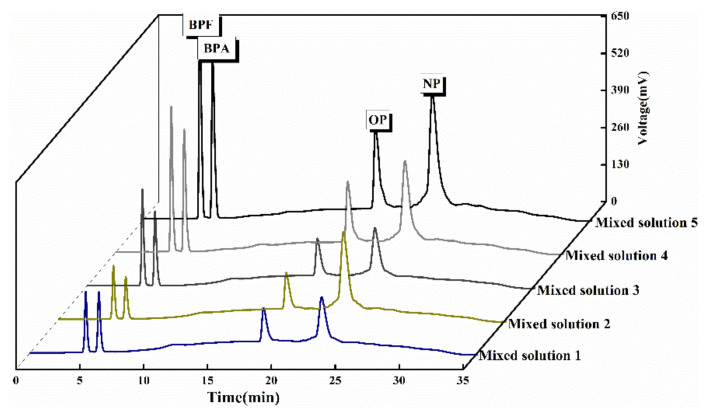
Standard curve of mixed substances.

As shown in Figure 9, it is the standard curve of mixed substances. It can be seen from the results in Table 3 that SPE-HPLC has a good linear relationship in the analysis and detection range of the four EDCs. The correlation coefficients of all analytes were greater than 0.99, and the relative standard deviation (RSD) was less than 1%, indicating that the analytical method can meet the requirements of trace detection of phenolic interferents in food.

#### 3.5.2. Comparison of Material Properties

Table 4 compared this experiment with the reported SPE method. It was found that the recovery of this experiment was higher than those of the reported method when it was used for the detection of actual samples. Therefore, a new SPE method for EDCs detection was developed in this experiment and a new filler was provided for the detection.

#### 3.5.3. Precision Analysis of Four EDCs by Solid Phase Extraction

Precision refers to the degree of deviation between the measurement results of each experiment and the average value of the total measurement data, which was used to indicate the repeatability of the method. In experiments, relative standard deviation (RSD) was usually used to express the precision of analytical methods.

As shown in Table 5, the relative standard deviation of the two methods was less than 3%, that was, the solid phase extraction of four EDCs by X–A and X–C methods had good accuracy.

#### 3.5.4. Feasibility Analysis of Solid Phase Extraction of COF Materials

**Figure 10:**
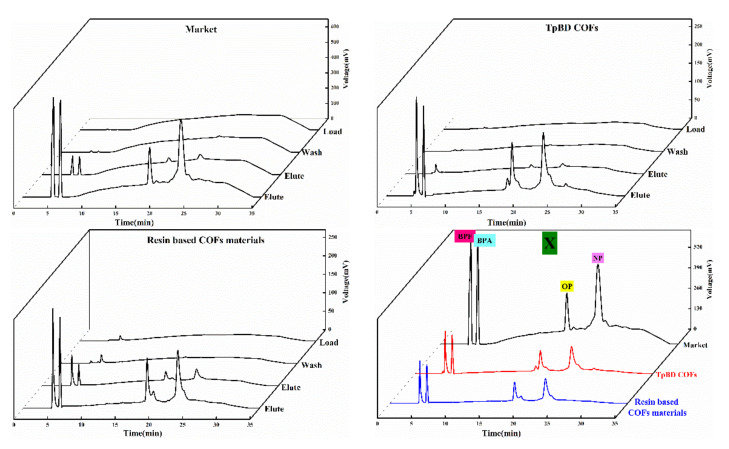
Outflow curves of four substances using the X method.

**Figure 11:**
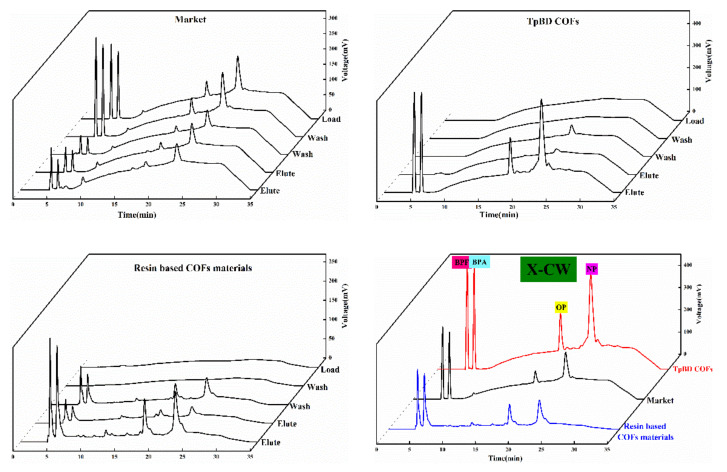
Outflow curves of four substances using the X-CW method.

**Figure 12:**
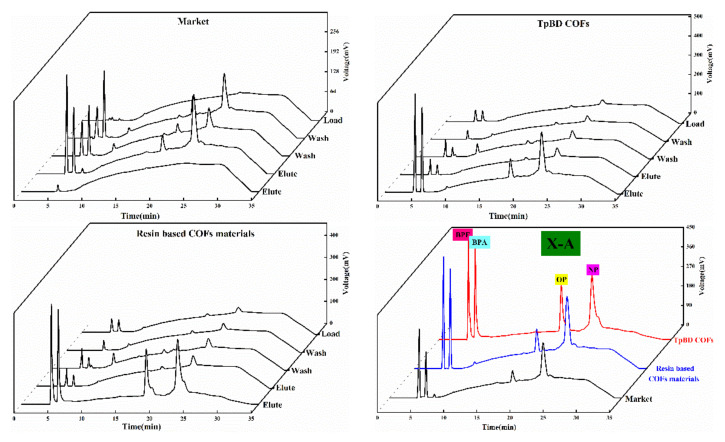
Outflow curves of four substances using the X-A method.

**Figure 13:**
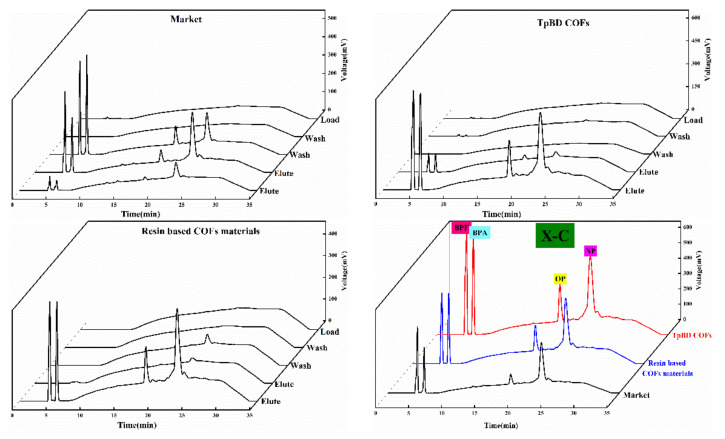
Outflow curves of four substances using the X-C method.

Four kinds of solid phase extraction schemes were selected in the experiment, and the adsorption and separation effects of the four kinds of phenol mixtures were investigated according to the extraction scheme in Table 1. The chromatographic outflow curves were showed in Figure 10, Figure 11, Figure 12 and Figure 13. Taking the retention time of the substance to be determined on SPE as the evaluation standard, through the comparison of the enrichment results of four phenols by commercial extraction column and self-made COF extraction column, it could be seen that the effects of X and X–CW schemes were not ideal. The SPE effect of X method was as follows: commercial extraction column > TpBD filler SPE column≈resin based COF material filler SPE column. In the case of X–CW method, When X-CW method was used, the effect was: TpBD filler SPE column>resin based COF material filler SPE column≈commercial extraction column. When X–A and X–C were selected, the solid phase extraction effect of TpBD COF and resin based COF were better than that of commercial solid phase extraction. The results proved that the solid phase extraction effect of X-A model was TpBD filler SPE column≈resin based COF material filler SPE column>commercial extraction column, and it could be seen from the solid phase extraction effect that the adsorption and separation effect of the new material as solid phase extraction filler was similar to that of other COF materials, which indicated that the prepared new COF material had good performance. The results showed that the solid phase extraction efficiency of X-C model was TpBD filler SPE column>resin based COF material filler SPE column>commercial extraction column. What is more, it could be seen that when X-A and X-C extraction schemes were used, the outflow time of samples was generally in the elution step, while X and X-CW extraction schemes had a certain outflow of samples at each stage. This phenomenon further indicated that X-A and X-C schemes were superior than X and X-CW schemes in solid phase extraction experiments.

In addition, the effect of solid phase extraction was analyzed from the perspective of solvent and COF material. In the extraction experiment, the solvent with smaller polarity was usually selected for washing, because if the high polarity solvent was selected, the target substance would have a certain solubility and compete with the adsorbent, which would destroy the polar force between the adsorbent and the target substance, resulting in the adsorbent could not be effectively adsorbed. Therefore, both X-A and X-C methods selected methanol solution with smaller polarity. In addition, weak acid and weak base ammonium acetate were added in the X-A scheme, which could enhance the ionic strength and reduce the solubility of polar organic compounds in water, That is to say, it played the role of salting out, so that the adsorbent could absorb more analytical components and effectively improve the extraction efficiency. In the X-C scheme, hydrochloric acid solution was used to adjust the pH value of the liquid, which could prevent the separation of analytical components and improve the adsorption capacity of the stationary phase. In the process of elution, the polar force between the adsorbent and the target compound was destroyed by high polar solvent. At the same time, high ion concentration could also destroy the polar interaction force, so in the elution step, X-A and X-C schemes were used to investigate the influence of elution solvent on pH. Through the analysis of the experimental results, it could be seen that the solid phase extraction effect of X-C scheme was better. Therefore, in the elution step, X-A and X-C schemes investigated the influence of eluent solvent on pH. through the analysis of the experimental results, it could be seen that the solid phase extraction effect of X-C scheme was better. Therefore, it could be concluded that the recovery rate of the target substance in the solid phase extraction experiment using resin based COF as solid phase extraction filler was higher than that of other methods. Generally, the effect of the target compound on the adsorbent depended on the interaction between the polar functional groups of the target compound and the polar functional groups on the surface of the adsorbent, i.e., hydrogen bond, *π-π* bond interaction and dipole–dipole interaction. For hydrogen bonding, because COF materials had *H* which was connected with atoms (*O, N*) with large electronegativity and small radius, and the polarity of hydrogen oxygen bond was very strong, the shared electron pair would be biased toward oxygen, at this time, the oxygen with partial negative charge of another water molecule was close, resulting in hydrogen bonding; for *π-π* interaction, because it existed in some compounds with conjugated structure, so it was also the main driving force of molecular self-assembly. This feature also enabled COF materials to adsorb target compounds effectively. In addition, for the dipole–dipole interaction, the non-uniform charge distribution of COF material was utilized to adsorb the target compound, and the unique interactions of these COF materials were well utilized in the SPE experiment. Analysis demonstrated that the above would homemade COF materials (resin base COF material and TpBD COF) as solid phase extraction adsorbents and adopted two kinds of schemes of Table 1 of phenols endocrine disruptors in drinks extraction separation had good extraction effect, and homemade resin base COF material could be used as solid phase extraction adsorbents to replace commercialization of solid phase extraction column and its economic cost in the application for adding the cheap resin base material was lower than the COF material had appeared.

#### 3.5.5. Analysis of the Results of Solid Phase Extraction of Phenols in Beverages

**Figure 14:**
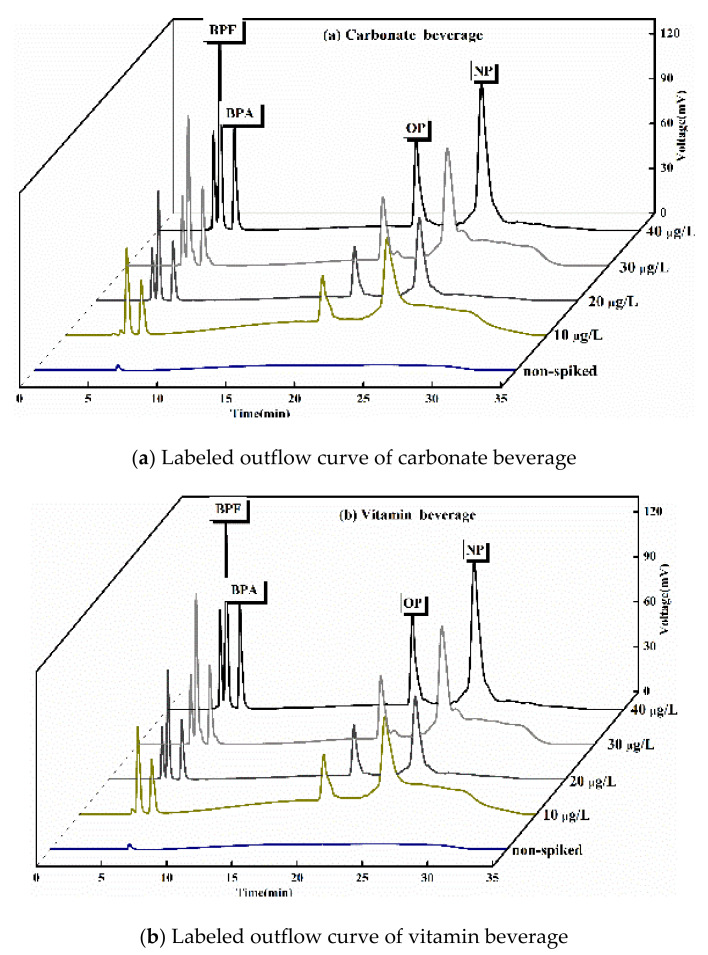
Labeled outflow curves of two kinds of beverages. (**a**) Labeled outflow curve of carbonate beverage, (**b**) Labeled outflow curve of vitamin beverage.

Figure 14 shows the outflow curve of the two drinks when the mixed solution with different concentrations was added. The results confirmed that the COF-SPE-HPLC method was suitable for the analysis of the beverage samples. Table 6 shows the experimental results of SPE recovery of four phenolic interferences when TpBD COF and resin based COF were used as SPE adsorbents for the two beverage samples. The recoveries of phenolic interferers in carbonated beverages and vitamin beverages were 98.18–102.18% and 98.52–101.79%, respectively. The analysis of the experimental results showed that four phenolic compounds were extracted by using two kinds of self-made COF materials as solid phase extraction fillers, the recovery rate of disruptors meet the requirements for the detection of trace concentrations of pollutants in environmental samples analysis, both COF can be used as solid phase extraction fillers in the enrichment, detection and analysis of trace EDCs in beverages and foods, and showed good anti-interference ability.

#### 3.5.6. Reusability of Materials

**Figure 15:**
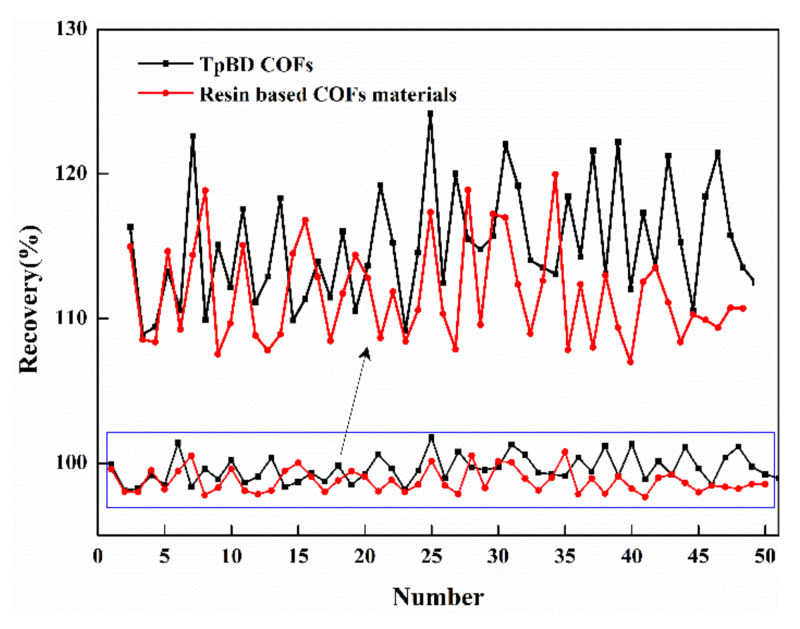
Recovery rate of solid phase extraction of materials.

As shown in Figure 15, the experiment was carried out in 50 repeated experiments, and it was found that the recovery rate of the two materials did not decrease significantly after the solid phase extraction experiment, indicating that the two materials had good binding force and stable performance, and the material could be reused in the experiment. Could be found in the contrast, TpBD COF material recovery rate was higher than that of the resin based COF material, but the recovery rate of two materials was between 97–101%, and the volatility of the recovery was 3.69% and 2.96%, respectively, demonstrated that the performance of the two materials were good and suitable for solid phase extraction experiments, but from the perspective of cost, resin based COF material cost was much lower than TpBD COF, and there was no significant difference between performance and application effect, the preparation of new material was more suitable for industrial production.

## 4. Conclusions

Two kinds of covalent organic frameworks (TpBD COF and resin based COF were prepared), and their structures were characterized. The feasibility of using COF as solid phase extraction (SPE) fillers to detect four EDCs (BPF, BPA, OP and NP) in beverages and foods was investigated.

(1) Two kinds of COF (TpBD COF and resin based COF) could be prepared by hydrothermal method with trialdehyde phloroglucinol as the synthetic monomer. The prepared COF had abundant pores, large specific surface area (TpBD COF: 814.6 m^2^/g; resin based COF: 623.9 m^2^/g) and good thermal stability.

(2) Two kinds of COF materials were applied to the solid phase extraction of EDCs in beverage samples. The precision RSD of this method was less than 3%. The recoveries were 98.18–102.18% and 98.52–101.79%, respectively. In addition, the recovery rate of the material did not change significantly in the 50 cycles of solid phase extraction, indicating that the material could be recycled and it could meet the requirements of the detection and analysis of trace pollutants in environmental samples. Therefore, the two kinds of COF materials had ideal enrichment and separation effect on the four kinds of phenolic disruptors, and the anti-interference ability of the materials was strong, and the extraction effect was better than the four kinds of commercial solid phase extraction columns.

(3) After COF materials were combined with resin, the product quantity of resin based COF was eight times of that of COF material. This method greatly reduced the preparation cost of COF material, and provided a new way for preconcentration and determination of trace harmful substances in beverage samples using COF materials as high performance solid phase extraction adsorbent.

## Figures and Tables

**Table 1:** Four SPE schemes.

Model	Active	Load	Wash	Elute	Elute
X	1 mL methanol	1 mL diluted mixed sample	1 mL 50% methanol	--	2 × 500 μL methanol containing 2% formic acid
TpBD COF					
Resin based COF material					
X-CW			1 mL water	1 mL methanol	2 × 500 μL methanol containing 5% formic acid
TpBD COF					
Resin based COF material					
X-A			1 mL methanol containing 25 mM ammonium acetate	1 mL methanol	2 × 500 μL methanol containing 5% formic acid
TpBD COF					
Resin based COF material					
X-C			1 mL methanol containing 0.1 N hydrochloric acid	1 mL water containing 0.1 mol/L HCl	2 × 500 μL methanol containing 5% ammonia
TpBD COF					
Resin based COF material					

**Table 2:** Specific surface area properties of COF materials.

COF	Specific Surface Area ^a^ (m^2^g^−1^)	Pore Size ^b^ (nm)	Pore Volume ^c^ (mL g^−1^)
TpBD COF	814.6	8.2	0.20
Resin based COF material	623.9	25.4	0.14

a: according to the Brunauer–Emmett–Teller (BET) formula; b,c: calculated according to Barrett–Joyner–Halenda (BJH) formula.

**Table 3:** Linear relationship and detection limit of phenols.

EDCs	Linear Regressive Equation	R^2^	RSD(%) n = 5
BPF	y = 1.78 × 10^7^ × x + 4.16 × 10^5^	0.9959	0.484
BPA	y = 5.81 × 10^7^ × x − 6.19 × 10^5^	0.9926	0.148
OP	y = 5.54 × 10^7^ × x − 1.84 × 10^6^	0.9989	0.365
NP	y = 1.09 × 10^8^ × x − 6.37 × 10^6^	0.9924	0.561

**Table 4:** Comparison with the reported EDCs determination method [20,22,23,24,25].

Sorbent	Method	Sample	Elution Solution	Recovery	Ref.
COF	SPE-HPLC	Beverage and milk	acetonitrile	82.0–96.3%	20
Oasis HLB	LC-MS	River water and industrial effluents	ammonia-methanol	83.1–108.4%	22
Fe3O4@MgAl-LDHs	MSPE-HPLC	Fruit juice, tea drink and soda	menthol	84.4–101.3%	23
HLB	SPE-HPLC	Water	methanol	89.7%~109.2%	24
Fe3O4@MON-NH2	MSPE-HPLC	water, beverage bottle and juice	methanol	80.3–109.5%	25
Self made materials	SPE-HPLC	beverage	methanol	98.18–102.18%	This work

**Table 5:** Results of solid phase extraction of four kinds of endocrine disruptors.

Analyte	Method	Peak Area (×10^6^)						Average (×10^6^)	SD (×10^6^)	RSD(%)
Mixture1	X–C	BPF	3.13	2.96	3.03	3.04	3.04	3.040	0.0604	1.99
		BPA	1.23	1.18	1.18	1.20	1.19	1.195	0.0208	1.74
		OP	1.95	2.00	1.98	2.02	2.03	1.996	0.0321	1.61
		NP	5.19	4.94	5.19	5.04	5.05	5.082	0.1076	2.12
	X–A	BPF	2.74	2.62	2.68	2.71	2.80	2.710	0.0671	2.48
		BPA	1.34	1.31	1.32	1.32	1.32	1.320	0.0112	0.85
		OP	3.60	3.67	3.73	3.51	3.70	3.642	0.0881	2.42
		NP	5.09	4.72	4.92	4.86	4.86	4.89	0.1338	2.74
Mixture2	X–C	BPF	5.42	5.33	5.40	5.36	5.40	5.384	0.0364	0.68
		BPA	3.18	3.27	3.22	3.26	3.30	3.243	0.0468	1.44
		OP	6.27	6.15	6.14	6.20	6.17	6.186	0.0522	0.84
		NP	11.34	11.50	11.14	11.06	10.99	11.206	0.2073	1.85
	X–A	BPF	4.96	4.80	4.98	4.91	4.89	4.908	0.0706	1.44
		BPA	3.04	3.22	3.08	3.13	3.16	3.124	0.0699	2.24
		OP	7.91	7.90	7.68	7.81	7.87	7.830	0.0946	1.21
		NP	14.25	14.14	13.51	13.99	14.06	13.989	0.2852	2.04

**Table 6:** Analysis of labeled recovery of beverage samples.

Sample	Method	Material	Targets	Spiked (μg/L)	Recovery (%, n = 3)
Carbonate beverage	X-A method	TpBD COF	BPF	10	99.67
				20	102.18
				30	98.83
				40	99.39
			BPA	10	99.63
				20	99.29
				30	100.27
				40	101.21
			OP	10	100.38
				20	99.44
				30	98.51
				40	99.28
			NP	10	99.45
				20	100.75
				30	99.58
				40	101.78
		Resin based COF material	BPF	10	99.63
				20	99.29
				30	100.27
				40	101.21
			BPA	10	100.38
				20	99.44
				30	98.51
				40	99.28
			OP	10	99.45
				20	100.75
				30	99.58
				40	101.78
			NP	10	99.92
				20	99.44
				30	100.73
				40	98.56
	X-C method	TpBD COF	BPF	10	98.24
				20	98.8
				30	98.9
				40	98.18
			BPA	10	100.01
				20	99.59
				30	100.29
				40	99.94
			OP	10	99.9
				20	93.12
				30	98.24
				40	99.17
			NP	10	98.52
				20	101.42
				30	98.36
				40	99.61
		Resin based COF material	BPF	10	98.9
				20	100.2
				30	98.65
				40	99.08
			BPA	10	100.38
				20	98.35
				30	98.71
				40	99.33
			OP	10	98.73
				20	99.83
				30	98.51
				40	99.26
			NP	10	100.6
				20	99.64
				30	98.18
				40	99.48
Vitamin beverage	X-A method	TpBD COF	BPF	10	101.79
				20	98.97
				30	100.79
				40	99.70
			BPA	10	99.53
				20	99.75
				30	101.28
				40	100.59
			OP	10	99.35
				20	99.23
				30	99.12
				40	100.41
			NP	10	99.41
				20	101.17
				30	99.12
				40	101.32
		Resin based COF material	BPF	10	98.87
				20	100.14
				30	99.22
				40	101.09
			BPA	10	99.65
				20	98.52
				30	100.41
				40	101.14
			OP	10	99.77
				20	100.38
				30	98.83
				40	98.79
			NP	10	100.30
				20	99.00
				30	100.24
				40	101.31
	X-C method	TpBD COF	BPF	10	98.59
				20	99.1
				30	100.4
				40	98.9
			BPA	10	89.65
				20	98.92
				30	100.26
				40	100.82
			OP	10	99.87
				20	98.81
				30	99.6
				40	100.24
			NP	10	99.86
				20	98.86
				30	99.63
				40	98.8
		Resin based COF material	BPF	10	99.32
				20	100.95
				30	99.26
				40	98.67
			BPA	10	101.32
				20	99.08
				30	100.92
				40	100.86
			OP	10	99.75
				20	98.93
				30	99.81
				40	101.58
			NP	10	98.66
				20	99.75
				30	98.7
				40	99.9

## Data Availability

The data presented in this study are available on request from the corresponding author.
